# Intraoperative serosal extracellular mapping of the human distal colon: a feasibility study

**DOI:** 10.1186/s12938-021-00944-x

**Published:** 2021-10-16

**Authors:** Anthony Y. Lin, Chris Varghese, Peng Du, Cameron I. Wells, Niranchan Paskaranandavadivel, Armen A. Gharibans, Jonathan C. Erickson, Ian P. Bissett, Greg O’Grady

**Affiliations:** 1 Department of Surgery, Faculty of Medical and Health Sciences, University of Auckland, Private Bag 92019, 1142 Auckland, New Zealand; 2 Auckland Bioengineering Institute, University of Auckland, Auckland, New Zealand; 3 Department of Physics-Engineering, Washington & Lee University, Lexington, VA USA; 4 Department of Surgery, Auckland City Hospital, Auckland, New Zealand

**Keywords:** Slow-wave, Colonic, High-resolution, Motility, Colonic physiology, Electrical mapping

## Abstract

**Background:**

Cyclic motor patterns (CMP) are the predominant motor pattern in the distal colon, and are important in both health and disease. Their origin, mechanism and relation to bioelectrical slow-waves remain incompletely understood. During abdominal surgery, an increase in the CMP occurs in the distal colon. This study aimed to evaluate the feasibility of detecting propagating slow waves and spike waves in the distal human colon through intraoperative, high-resolution (HR), serosal electrical mapping.

**Methods:**

HR electrical recordings were obtained from the distal colon using validated flexible PCB arrays (6 × 16 electrodes; 4 mm inter-electrode spacing; 2.4 cm^2^, 0.3 mm diameter) for up to 15 min. Passive unipolar signals were obtained and analysed.

**Results:**

Eleven patients (33–71 years; 6 females) undergoing colorectal surgery under general anaesthesia (4 with epidurals) were recruited. After artefact removal and comprehensive manual and automated analytics, events consistent with regular propagating activity between 2 and 6 cpm were not identified in any patient. Intermittent clusters of spike-like activities lasting 10–180 s with frequencies of each cluster ranging between 24 and 42 cpm, and an average amplitude of 0.54 ± 0.37 mV were recorded.

**Conclusions:**

Intraoperative colonic serosal mapping in humans is feasible, but unlike in the stomach and small bowel, revealed no regular propagating electrical activity. Although sporadic, synchronous spike-wave events were identifiable. Alternative techniques are required to characterise the mechanisms underlying the hyperactive CMP observed in the intra- and post-operative period.

**New findings:**

The aim of this study was to assess the feasibility of detecting propagating electrical activity that may correlate to the cyclic motor pattern in the distal human colon through intraoperative, high-resolution, serosal electrical mapping. High-resolution electrical mapping of the human colon revealed no regular propagating activity, but does reveal sporadic spike-wave events. These findings indicate that further research into appropriate techniques is required to identify the mechanism of hyperactive cyclic motor pattern observed in the intra- and post-operative period in humans.

**Supplementary Information:**

The online version contains supplementary material available at 10.1186/s12938-021-00944-x.

## Introduction

Colonic motility disorders are common [1–5], and are often classified as functional disorders on the basis of symptoms alone after excluding an anatomical cause, medication effect, or systemic illness [6]. The pathophysiology of these disorders remains incompletely understood, contributing to challenges in developing effective diagnostics and targeted treatments. Electrophysiological abnormalities may play a role in colonic motility disorders, but the precise characteristics of the electrical activity resulting in dysmotility largely remain unclear.

High-resolution (HR) colonic manometry has recently revealed the cyclic motor pattern (CMP) to be the dominant motor pattern of the distal colon [7]. CMPs, which arise or terminate at the functional sphincter of O’Beirne at the rectosigmoid junction [8], are thought to be responsible for the ‘rectosigmoid brake’ [9, 10], acting to limit rectal filling and contributing to the control of continence [9, 10]. Altered CMPs have been implicated in the pathophysiology of constipation [11, 12] and low anterior resection syndrome [13]. Hyperactive CMPs have previously been observed intraoperatively while patients are under general anaesthesia for abdominal surgery, potentially representing a novel effect of the surgical stress response [14, 15]. CMPs are also a target of emerging diagnostics such as high-resolution body surface mapping [16, 17], and may be a relevant biomarker for therapeutic sacral neuromodulation [18]. However, despite a broad emerging diagnostic and therapeutic significance, further work is required to better characterise the electrophysiological origins of the CMP.

Gut peristalsis is coordinated by several cooperating mechanisms [19], and one such mechanism that is postulated to play a contributing role in the CMP are slow-waves generated by networks of interstitial cells of Cajal (ICC) operating in concert with enteric, autonomic and myenteric factors [9]. Two slow waves frequency ranges are commonly described in the colon, one at 2–6 cpm and one at 9–14 cpm [20–23]. Additionally, ‘spike waves’ have also been recorded in the colon, representing a travelling sequence of spike-like events that occur independently or at the plateau of slow waves and reflect smooth muscle activation [24–26].

The electrical activity in the human colon is poorly understood, and more difficult to measure [27, 28]. Further insights into the electrophysiological activity of the distal colon will require in vivo measurements. Novel technical approaches are required to resolve the colonic electrical activity underpinning now well-established motility patterns on manometry. In particular, novel mechanistic insights are needed to better direct future research into the aetiology of colonic motility disorders, such as post-operative ileus and slow-transit constipation, and to direct neuromodulation strategies [11, 14, 18]. This study, therefore, aimed to assess the feasibility of performing intraoperative, high-resolution electrical mapping of the distal colon’s electrical activity.

## Results

**Fig. 1:**
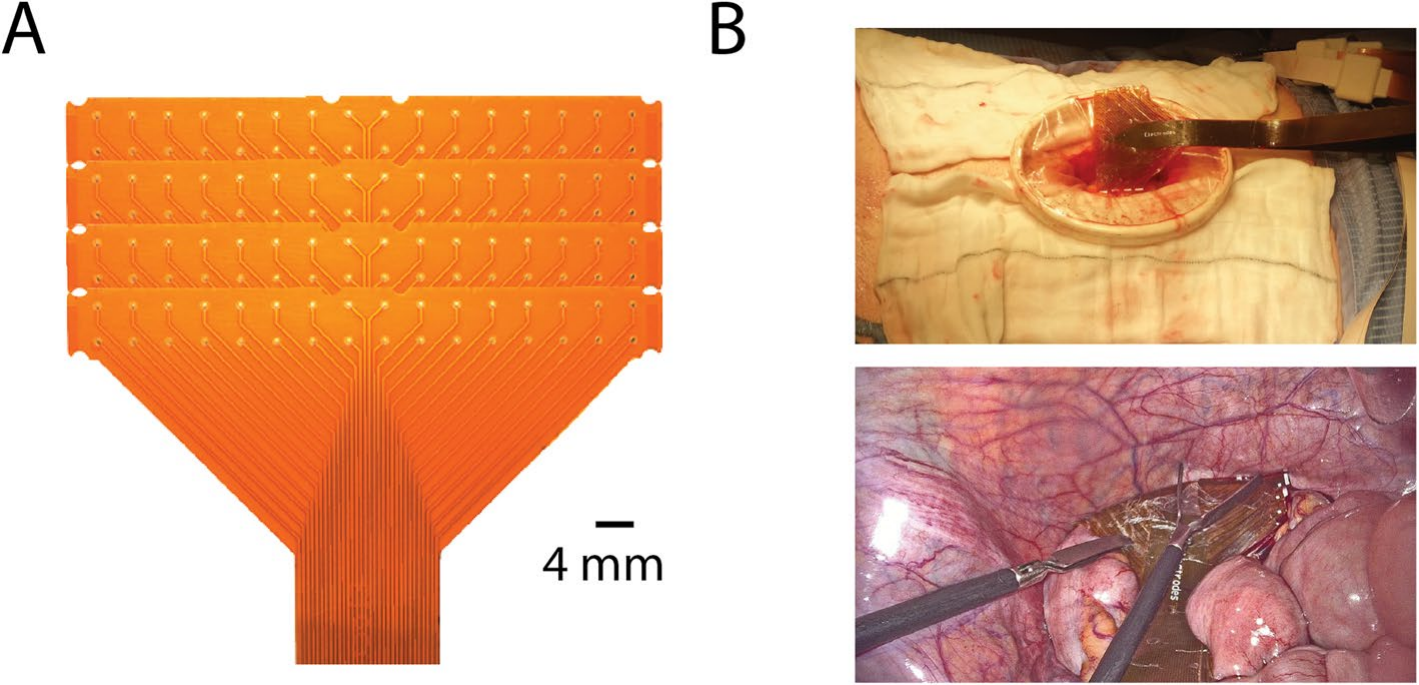
HR electrical recording using intraoperative placement of multielectrode arrays. **A** The tessellated FPC electrode array (4 × 32 electrode; 4 mm inter-electrode spacing). **B** Top: insert of the FPC array through an Alexis wound retractor (Applied Medical, Rancho Santa Margarita, CA); bottom: laparoscopic placement of the flexible electrode array on the serosa of the colon

Eleven patients were recruited for the study (age 33–71 years; 5 M/6F). The patient demographics, indication for surgery and type of surgery are listed in Table 1. A total of 19 recording segments were taken (mean, 1.7 segments per patient) for a total duration of 4,895 s (s) (mean, 445 ± 136 s per patient and 257 ± 98 s per segment). Figure 1 shows an example of the placement of the multielectrode arrays. All recordings were taken at the end of operations after the completion of the resection and anastomosis, except for two cases where the recording was taken before the resection (Patient 8, sigmoid colectomy; and Patient 9, appendicectomy).

**Table 1:** Patient demographics and clinical details

Patient	Age and gender	Type of surgery	Primary pathology	Mode of anaesthesia
1	71F	Open APR	Anal SCC	General + epidural
2	72F	Open RH	Caecal cancer	General
3	75M	Lap RH	Ascending colon cancer	General
4	33M	Lap extended RH	Transverse colon cancer	General
5	69F	Open RH	Metastatic endometrial cancer	General + epidural
6	42M	Lap RH	Large hepatic flexure polyp	General
7	59F	Lap RH	Transverse colon cancer	General
8	66F	Open sigmoid colectomy + distal pancreatectomy	Metastatic endometrial cancer	General + epidural
9	55M	Lap appendicectomy	Appendiceal mucocele	General
10	53M	Open RH + duodenal wedge	Crohn’s stricture	General + epidural
11	68F	Lap RH	Caecal cancer	General

*Lap* laparoscopic, *APR*  abdominoperineal resection, *RH * right hemicolectomy, *SCC* squamous cell carcinoma

A detailed visual inspection of all raw and filtered data did not yield colonic activity which demonstrated propagation, delayed repolarisation, and a frequency consistent with that of the CMP. Application of a Butterworth filter with a passband of 10–600 cycles per minute (cpm) without additional baseline removal to all the data showed higher frequency components resembling spike waves.

**Fig. 2:**
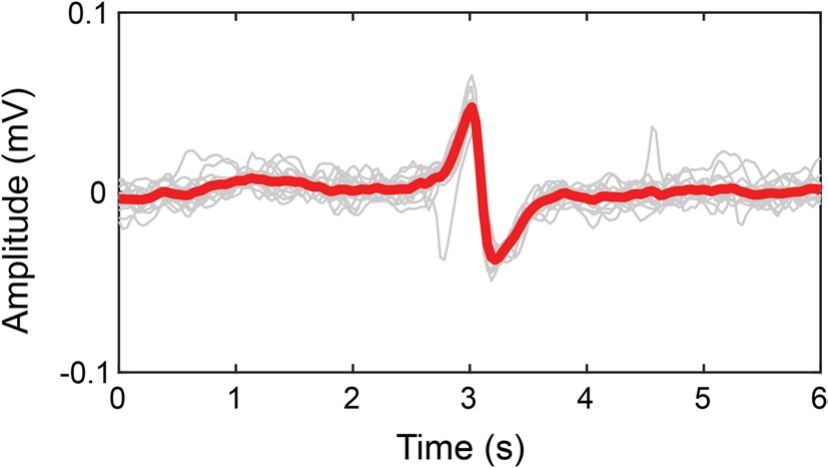
Average colonic spike waves recorded at the rectosigmoid region of the colon from 17 waves

The time–frequency analysis of patients’ spike event activity can be found in the Additional file 1: Appendices S1 and S2; these results are summarised in Table 2. No regular propagating activity with a fast-Fourier transform (FFT) cluster at ~ 3 cpm was seen in any patient. One 20-s window of activity was visualised in 5 channels at the sigmoid colon of Patient 7 that had a biphasic potential with a prolonged recovery phase, and apparent propagation at a frequency of ~ 15 cpm, however, with irregular depolarisation activity (Additional file 1: Figure S1). The average morphology of the colonic activity is shown in Fig. 2.

**Table 2:** Characteristics of bioelectrical signals recorded in the human colon intraoperatively

Patient	Regular propagating events	Spike events	Number of spike event bursts, n	Frequency, cpm mean (SD)	Amplitude, µV mean (SD)
1	✗	✗	0	–	–
2	✗	✗	0	–	–
3	✗	✗	0	–	–
4	✗	**✓**	26	18.69 ± 3.68	532.81 ± 117.19
5	✗	**✓**	15	15.56 ± 5.54	446.04 ± 154.71
6	✗	**✓**	4	14.47 ± 2.63	1277.76 ± 1698.44
7	**✓**	**✓**	28	15.91 ± 2.82	414.10 ± 159.27
8	✗	**✓**	35	8.97 ± 4.31	416.05 ± 282.39
9	✗	✗	6	12.28 ± 4.30	1992.73 ± 637.89
10	✗	**✓**	23	11.21 ± 3.46	744.51 ± 345.45
11	✗	**✓**	22	7.82 ± 5.51	444.60 ± 210.01

✓ represents the presence of the event-type described in the column header in the patient’s serosal extracellular recordings

### Periodic synchronous waveforms

**Fig. 3:**
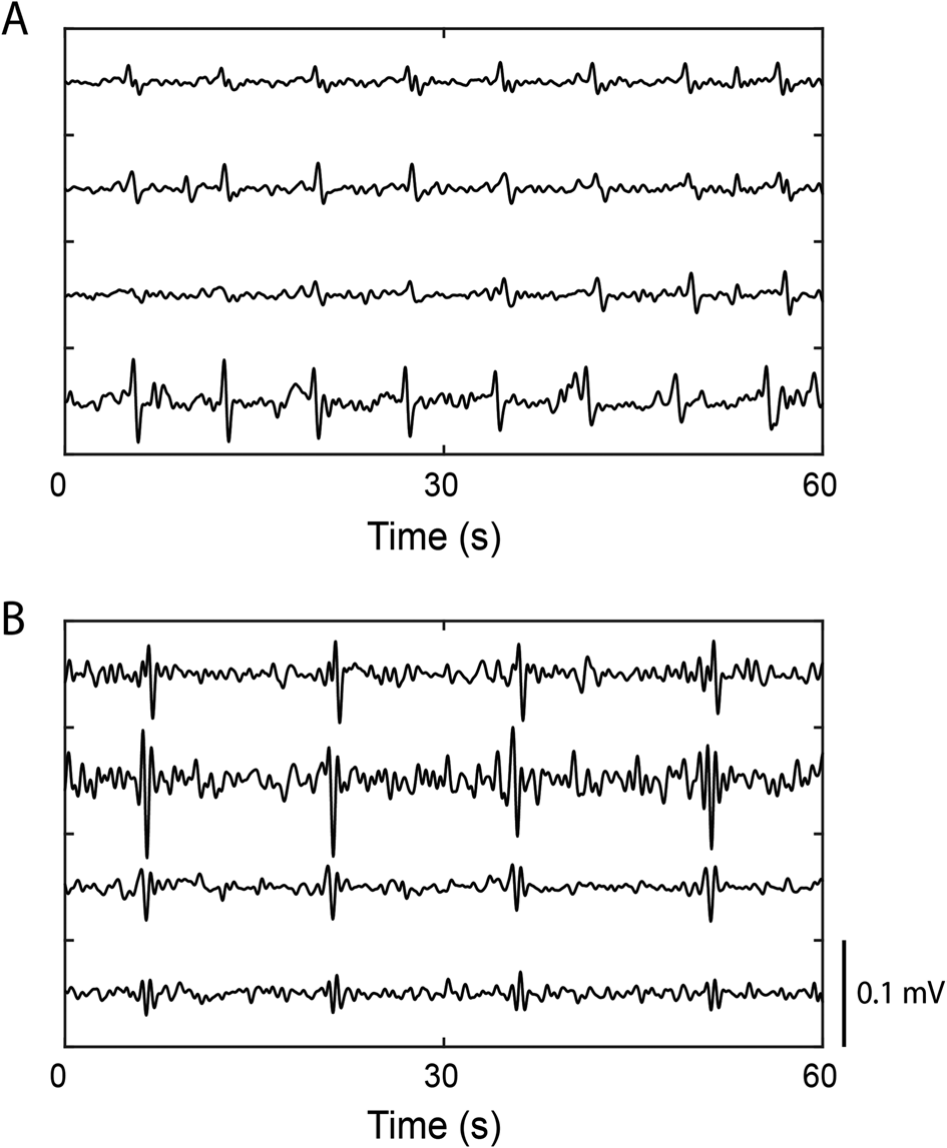
Periodic bioelectrical activity recorded from adjacent consecutive electrodes on the distal human colon. **A** 8 cpm activity. **B** 4.5 cpm activity. These were not considered propagating slow waves per pre-specified criteria, i.e. the simultaneous registration across multiple channels (no lag), morphology, and lack of slow wave repolarisation [29, 30]

In Patient 5 at the rectosigmoid junction, 4.5 cpm non-propagating electrical activity was seen synchronously across four channels at 0.13 ± 0.06 millivolts (mV) and another 8 cpm non-propagating electrical activity was seen synchronously across six channels at 0.05 ± 0.02 mV (Fig. 3). This rapid or non-propagating nature is consistent with other recordings of spike waves [24], and with simultaneous CMPs previously described in the human colon intraoperatively [14]. This activity was biphasic, and on occasion triphasic, but its morphology lacked the expected delayed repolarization typical of slow waves recorded elsewhere [29, 30].

### Variable spike-like activity

**Fig. 4:**
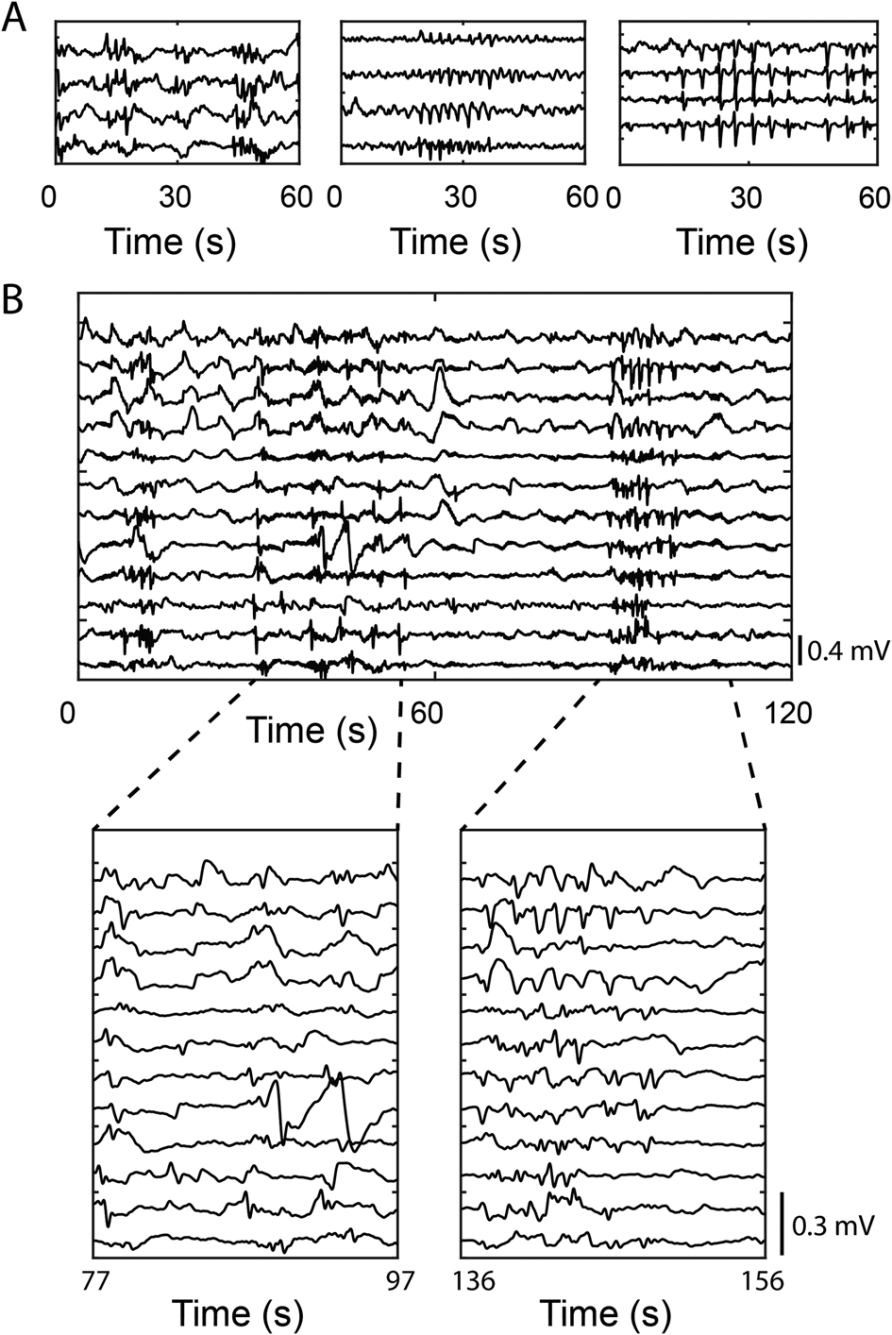
**A** Recording demonstrating three clusters of spike-like activity from one recording. **B** Morphology of spike-like activity within burst activity

Activities resembling irregular spike-like activity were seen in three patients. These were identified only in a few isolated channels and insufficient data were available to construct activation maps. The spike-like activity frequency ranged from 24 to 42 cpm, lasting 10–180 s with an average amplitude of 0.54 ± 0.37 mV (Fig. 4).

Recordings of other physiological data were obtained at times, including ventilator and cardiac parameters, according to the descriptors of Paskaranandavadivel et al. [31, 32]. The frequencies of these activities were consistent with the physiological range recorded on the anaesthetic monitor. Examples of artefact data can be found in Additional file 1: Figure S2 which demonstrate distinct activity patterns to the spike-like activity seen in Fig. 4.

Table 3 describes a comparison of colonic spike activity with small intestinal spike waves which reflect smooth muscle contraction. While morphological similarities exist, these activities differ in duration, frequency, burst patterns, and amplitude.

**Table 3:** Small intestinal spike waves compared to colonic spike burst activity

Features	Small intestinal spike activity*	Colonic spike burst activity
Duration (s)	0.5–1 s	10–180 s
Event frequency (Hz or cpm)	~ 10 Hz (~ 600 cpm)	24–48 cpm
Burst frequency	6–12/min	Sporadic/random (< 3/min)
Amplitude	50–100 uV	400–2000 uV
Morphology	Biphasic/variable	Biphasic; regular activity (*n* = 2); irregular/sporadic activity (*n* = 5)
Propagation	Short-durations or absent	Absent

*The small intestine spike activity metrics was extracted from study by Erickson et al. [26] with permission

## Discussion

This feasibility study reports a novel attempt to achieve serosal intraoperative HR electrical mapping of the human colon, using previously validated technical methods, in 11 human patients. This technical approach provides a robust and validated approach to evaluate colonic electrical activity. The selected colonic region was known to be mechanically active with cyclic motor patterns present > 50% of the time during surgery [14], such as to present a suitable context for the detection of any propagating slow wave patterns in humans [14]. Intraoperative, high-resolution serosal mapping demonstrated electrical activity between 7 and 19 cpm. Electrical activity was variable, rarely propagating and most frequently synchronous. Both regular and irregular spike-wave patterns were also present. Propagating patterns at a frequency range of 2–6 cpm consistent with the cyclic motor pattern were not recorded.

Periodic bursts of electrical activity were seen regularly in several patients; these spike bursts lasted 10–180 s and occurred at frequencies between 24 and 42 cpm. These events resembled spike waves at various frequencies. However, these spike waves have notable differences to those recorded in the small intestine, as described by Erickson et al. [26]. While small intestine spike bursts lasted 0.5 s and occurred at much higher frequencies ~ 10 Hertz (Hz) (~ 600 cpm); colonic spike events persisted for longer durations and at lower frequencies. The colonic spike activity appears more regular and rhythmic than is seen in the small intestine, reflecting physiological differences in smooth muscle contraction dynamics between the small and large intestine. Additionally, their simultaneous registration across the electrode array, as has been seen in the small intestine, may be caused by the absence of a slow wave frequency gradient [33]. These activities may also reflect oscillatory electrical colonic activity and may originate from ICC; likely ICC-MY [34]. Differences in rectal and colonic spike activities have been postulated to reflect differences in the location of serotonin-4 subtype receptors [35, 36], but further work is required. Similarly, it remains unknown if the spike activity might represent a response to surgical stress.

A previous study using intraluminal electrical recording found activity corresponding to 2–3 cpm and 10–12 cpm, termed long spike bursts and short spike bursts, respectively [23]. The long spike bursts occurred at a frequency of 23 per hour, with action potentials occurring at a frequency of 2–3 cpm [23]. The short spike bursts occurred at a frequency of 12 cpm. It is plausible the regular spike activity recorded in the present study may correspond to the long spike bursts and the variable (both falling within the CMP frequency range) and the higher frequency spike-like activity corresponds to the short spike bursts [23]. It is also possible that the irregular, propagating activity recorded over 20 s in 1 patient (Additional file 1: Figure S1), could also correlate with the long spike bursts, but further work is required to corroborate this. However, as these recordings were low-resolution, the nature of propagation of these activities could not be characterised.

Spike waves have been recorded extracellularly in the feline small intestine and dog rectum [24, 25]. These studies found that spike waves were sporadic, they tended to propagate shorter distances before spontaneous termination [25], and in a more isotropic pattern, than slow waves. Spike waves also tended to occur during the plateau phase of a leading propagating slow wave [25]. In contrast, slow waves have also been recorded in the submucosal surface of dog colons in vitro [37]; it was found this activity originating from the ICC-SM propagated primarily circumferentially, and short longitudinal distances necessitating neural control to sequentially coordinate slow waves to produce peristaltic events [37].

The primary pacemaker for colonic slow waves are the interstitial cells of Cajal found in the submucosal layer (ICC-SM) which may still be the source of 2–6 cpm activity on manometry. Serosal recordings may be unable to record activity from the ICC-SM due to their depth within the colonic wall [34]. Serosal recordings may be more likely to detect activity arising at the myenteric plexus (in ICC-MY), previously described as being consistent in frequency with the ~ 12–20 cpm band [34]. This activity has previously been described as myenteric potential oscillations occurring between 16 and 20 events per minute and are more consistent with the findings of this study [34]. Further work exploring the ICC-SM activity in the colon is required. This may require HR intraluminal recordings similar to the methods of Bueno et al. [23]; such work may be facilitated by advances in HR EMG methods currently limited to the anorectum [38]. Three-dimensional electrode arrays such as those used in intracortical mapping, may also be an avenue for future recording arrays to provide recording from multiple colonic layers [39].

It is unclear whether the spike waves recorded at 4 and 8 cpm in the present study may directly underpin the CMP, but this is plausible. The perioperative period, in particular the intra- and post-operative periods are associated with the surgical stress response, and the associated sympathetic drive is accompanied by an increase in the CMP [14]. The activation of the parasympathetic pelvic splanchnic nerves is also the hypothetical mechanism for the efficacy of SNS in faecal incontinence and potentially LARS [9]. Hence current literature suggests a complex interplay between the sympathetic and parasympathetic nervous systems to regulate human rectosigmoid motility [9, 18, 40–42]. Therefore, the autonomic nervous system might have an important coregulatory role in modulating the 2–6 cpm gut motility pattern patterned ICC networks.

Low-resolution electrical recordings from the human colon have previously been reported on the serosal surface and from mucosal suction electrodes [43–46]. Sarna et al. used bipolar electrodes to show two frequency ranges in the human distal colon, with the lower frequency range (2–9 cpm) being the most dominant [45]. Sarna et al. found that rhythmic electrical activity was regularly observed, but this only sporadically translated to electrical activity associated with contractions. These were termed discrete electrical response activity and continuous electrical response activity depending on the duration of action [43, 45]. These events may correspond to the spike patterns seen in the present study (e.g. Figs. 2 and 4).

Most previous serosal recordings from the colon were obtained using a bipolar recording method [44], whereas the HR electrical mapping uses multiple monopolar recordings. Bipolar recordings have the advantage of higher signal-to-noise ratio, however, have limitations that mean it is (1) more difficult to morphologically identify the repolarization phase and (2) more sensitive to the relative position of the bipolar electrode pair relative to the direction of propagation of the wavefront [47, 48]. As a lag between wavefronts of adjacent channels is ideally used to classify propagation, bipolar recordings are at higher risk of failing to characterise the propagation and recovery morphology.

The recordings were taken intraoperatively with patients under general anaesthesia, which might have altered the expression of electrical activity in the colon, but is unlikely to have affected the recordability of slow waves [48]. However, at present, no other HR electrical mapping studies of the human colon exist, and current available technology requires laparotomy or laparoscopy for the placement of the multielectrode arrays on the intestinal serosal surface. Additionally, similar intraoperative approaches for HR serosal electrical mapping via multielectrode arrays have been validated and found to be reliable at routinely recording gastric and small intestine slow waves in humans, when performed by the same team using the same techniques and equipment [29, 49–51]. Therefore, technical factors likely do not explain the lack of similar propagating colonic activity. The CMP has been successfully recorded and characterised through HR manometry and body surface mapping techniques [7, 16], however these are not of the same character and physiology of the slow waves recorded at the serosa [50]. Simultaneous recordings using HR manometry and HR electrical mapping techniques, as well as less invasive methods of recording electrical activity may be helpful [17].

Several limitations exist in recording electrical activity from the human colonic serosa. The short duration of recordings and only partial cover of the rectosigmoid serosal surface leaves the possibility that further propagating activity may have fallen outside the temporal or spatial window of recording. However, it is notable that no clear electrical correlate was recorded over the course of 11 patients with several minutes of recording data and focused recording of the rectosigmoid junction where such activity is known to occur during surgery. Additionally, from a technical standpoint, the signal-to-noise ratio for extracellular slow waves is lower for the small intestine compared with the stomach [50, 51], and this is likely to also be true for the colon, making the detection of slow waves more challenging. The colon also has a highly curved geometry compared to the stomach, which has a larger, flatter, and firmer surface for optimal tissue electrode contact. Application of wet sterile gauze and laparoscopic graspers were used to maintain adequate contact. The development of more pliable electrodes or electrodes with improved adherence to colonic serosal surfaces may aid in the detection and characterisation of colonic electrical activities in future. However, no significant electrical artefacts reflecting loss of contact were seen, adding confidence to the reliability of the recordings.

## Conclusion

In conclusion, HR electrical mapping of the human colonic serosa is feasible but shows few organised propagating electrical activities, although, sporadic, synchronous spike waves were present. Intraoperative serosal mapping of the colon remains technically challenging, and further validation using simultaneous HR colonic manometry is required.

## Methods

This was an intraoperative electrophysiological study using HR multielectrode arrays to record and define colonic electrical activity in patients undergoing abdominal surgery. Inclusion in this study did not affect patients’ selection to undergo the proposed operation or the operation itself. Ethical approval was obtained from the Northern B Health and Disability Ethics Committee (16/NTB/196) and studies conformed to the standards set by the latest revision of the Declaration of Helsinki except registration in a database.

### Patients and controls

Patients undergoing abdominal intestinal operations, aged between 18 and 75 years, and able to provide informed consent were eligible for this study. The exclusion criteria included pregnancy; those with an American Society of Anaesthesiologists (ASA) score greater than or equal to 4; previous colorectal resection except for appendicectomy; and those with a significant metabolic, neurologic, or endocrine disorder known to cause dysmotility. Patients with abnormal bowel habits were also excluded.

### Sample size

This was the first human study applying HR electrical mapping to the colon. No existing data were available for power calculation, so numbers were estimated from previous successful mapping studies performed on the stomach and small intestine (10–12 patients per published cohort) [50–52].

### Study protocol

Patients followed a standard Enhanced Recovery After Surgery (ERAS) protocol used by the Colorectal Unit at Auckland District Health Board; in brief, no bowel preparation for right-sided colonic resections, preoperative carbohydrate drink, minimisation of intraoperative fluid use, and early postoperative feeding and mobilisation.

All patients fasted from midnight on the day of the procedure other than the preoperative carbohydrate drink and were given standardised premedication and perioperative analgesia. This protocol included (1) 1 g oral paracetamol preoperatively; (2) prophylactic antibiotics administered intravenously, typically cefuroxime and metronidazole; (3) benzodiazepine premedication, such as midazolam; (4) an epidural catheter selectively for those planned to undergo open surgery, typically employing ropivacaine or bupivacaine; (5) a short-acting intravenous opiate; (6) a muscle relaxant, typically suxamethonium or atracurium; (7) an anaesthetic induction agent; and (8) an inhalational agent such as isoflurane or sevoflurane. Other medications were used if clinically indicated. These anaesthetic conditions were similar to that of Vather et al.’s protocol [14, 50, 53] and were similar to those used in a recent high-resolution intraoperative manometry study where CMPs were found to be hyperactive during surgery [14].

All procedures were carried out under general anaesthesia. The anaesthetic protocol and the surgical approach were left to the discretion of the anaesthetic and surgical teams. The mode of anaesthesia was recorded and reported. Both laparoscopic and open surgical approaches were used, depending on the surgeon’s preference and the clinical indication.

### High-resolution electrical mapping

High spatial resolution electrical recordings were made via custom-made multielectrode, flexible printed circuit board (PCB) arrays (FlexiMap, New Zealand) [49]. There were 4 tessellated PCB arrays each containing 32 electrodes, of 0.3 mm diameter, printed over two rows at 4 mm intervals. The PCB arrays were made of polyamide with copper inlaid and gold-plated channels and had a thickness of ~ 0.08 mm. These have previously been used in measuring human gastric extracellular electrical signals [50]. The 128-channel, multielectrode arrays were positioned intraoperatively through an open incision or a laparoscopic port (known to record congruent data from previous intraoperative gastric recording experience [50]). Recordings were planned to be taken at the end of the operation after the completion of resections and/or anastomoses. Recordings were obtained from the serosal surface (antimesenteric side) of the colon at the rectosigmoid junction and sigmoid colon. Warm, moist gauze packs were placed in open operations to stabilise the multielectrode arrays. In laparoscopic cases, contact against the colonic serosal surface was applied via gentle application of laparoscopic graspers as previously described [54].

Passive, unipolar recordings were obtained from the ActiveTwo system (Biosemi, Amsterdam, Netherlands). Each multielectrode array was connected to the recording system via a sterilised 1.5 m 32-flat flexible cable, which was in turn connected via a fibre-optic cable to a computer. Reference electrodes were placed on the shoulder of each patient. Customised acquisition software written in LabView v8.2 (National Instruments, Austin, Texas, USA) was used. Once a satisfactory signal was obtained (free of interference), the recording continued for up to a maximum of 15 min that included 5 min where patients were left undisturbed. The ventilator was paused for a brief period of 30–60 s during the recording at the discretion of the anaesthetic team for a more stable recording. On several occasions, the multielectrode arrays were repositioned to improve the signals. This resulted in additional recording segments for some patients. The placement positions were recorded with intraoperative drawings. The multielectrode arrays were removed at the end of the recording before the end of the surgery. Sterility was maintained throughout insertion, recording and removal of the multielectrode arrays. All of these techniques were the same as previously used in human intraoperative gastric and small bowel studies where slow waves were successfully recorded (e.g. [50, 51, 54]).

### Data analysis

Data analysis was performed using the Gastrointestinal Electrical Mapping Suite (GEMS) v1.7 (FlexiMap, New Zealand) [55]. Raw data were visually inspected and analysed [48]. Thereafter, artefacts and noise were removed, and signals from all channels were analysed using activation maps to analyse the characteristics of the slow waves [48], and spike waves [26], with capability to map wave frequency, direction and propagation velocity vectors.

Raw data were down-sampled from 512 to 30 Hz after low-pass filtering to prevent aliasing of high-frequency signals. A low-pass Savitzky–Golay filter was used to smooth the recorded signals with a cut-off frequency of 1.98 Hz, which was previously applied in the analysis of 3 cpm gastric slow waves [31]. In the absence of consistent slow waves < 10 cpm, the data were filtered separately with Butterworth filter with a passband of 10–600 cpm without additional baseline-drift removal, and the higher frequency components (i.e. spike-like waves) were visualised in GEMS [56]. In each patient where spike events were recorded, the dominant frequency was calculated via a shifting fast-Fourier transform (FFT) with a 20-s window and a shift of 15 s. The results were plotted in a time–frequency plot, which depicts the amplitude of the dominant burst frequency. Classification of slow waves required prospective registration of biphasic depolarisations with a delayed repolarization phase within a 1–6 cpm range on time–frequency analysis. Slow waves also had to be propagating with a demonstrable time-lag between adjacent channels indicating propagation [48].

The morphology of recorded colonic activities was visualised by taking the average of a series of previously recorded intraoperative colon data from the present study. Data from ± 3 s on either side of an electrical event with deflection from baseline were taken and the time of the maximum amplitude was used to align the multiple waves before they were averaged.

### Statistical analysis

Frequency and amplitude data were expressed as mean ± SD. Statistical analysis was planned to be performed on the retrieved physiological patterns, however, the nature of the data retrieved in this exploratory study was insufficient to quantitatively interrogate the underlying electrical physiology.

## Supplementary Information

**Additional file 1: Appendix S1.** Individual patient spike event analysis. Segments where spike activity were present are shown with the most representative channel and a corresponding time–frequency plot. **Figure S1.** Patient 7 with ~ 15 cpm, irregular depolarisation activity with apparent propagation and prolonged recovery phase after each initial depolarisation. This was seen in 5 channels over a period of 20 s. **Appendix S2.** Artefacts. Recordings of other physiological data were obtained at times, including ventilator and cardiac parameters, according to the descriptors of Paskaranandavadivel et al. [31, 32]. The frequencies of these activities were consistent with the physiological range recorded on the anaesthetic monitor. Figure S2 shows several examples of these activities. **Figure S2.** Artefacts obtained on recordings. **A** Ventilator artefacts. **B** Cardiac activity.

## Data Availability

Data are available within the article and its supplementary materials.

## Abbreviations

HR: High-resolution
CMP: Cyclic motor pattern
SD: Standard deviation
Cpm: Cycles per minute
FFT: Fast-Fourier transform
Hz: Hertz
ICC: Interstitial cells of Cajal
ICC-SM: Interstitial cells of Cajal—submucosal
ICC-MY: Interstitial cells of Cajal—myenteric plexus
s: Seconds
mV: Millivolts
PCB: Printed circuit board
