# Supplementary material for: Intraoperative serosal extracellular mapping of the human distal colon: a feasibility study
Source: Biomed Eng Online. 2021 Oct 16;20:105. doi: 10.1186/s12938-021-00944-x (PMC8520224; doi:10.1186/s12938-021-00944-x)
Supplement: Supplementary file 1 — Additional file 1: Appendix S1. Individual patient spike event analysis. Segments where spike activity were present are shown with the most representative channel and a corresponding time–frequency plot. Figure S1. Patient 7 with ~ 15 cpm, irregular depolarisation activity with apparent propagation and prolonged recovery phase after each initial depolarisation. This was seen in 5 channels over a period of 20 s. Appendix S2. Artefacts. Recordings of other physiological data were obtained at times, including ventilator and cardiac parameters, according to the descriptors of Paskaranandavadivel et al. [31, 32]. The frequencies of these activities were consistent with the physiological range recorded on the anaesthetic monitor. Figure S2 shows several examples of these activities. Figure S2. Artefacts obtained on recordings. A Ventilator artefacts. B Cardiac activity. [file 12938_2021_944_MOESM1_ESM.docx]

**Additional file 1: Appendix S1:** Individual patient spike event analysis. Segments where spike activity were present are shown with the most representative channel and a corresponding time-frequency plot.


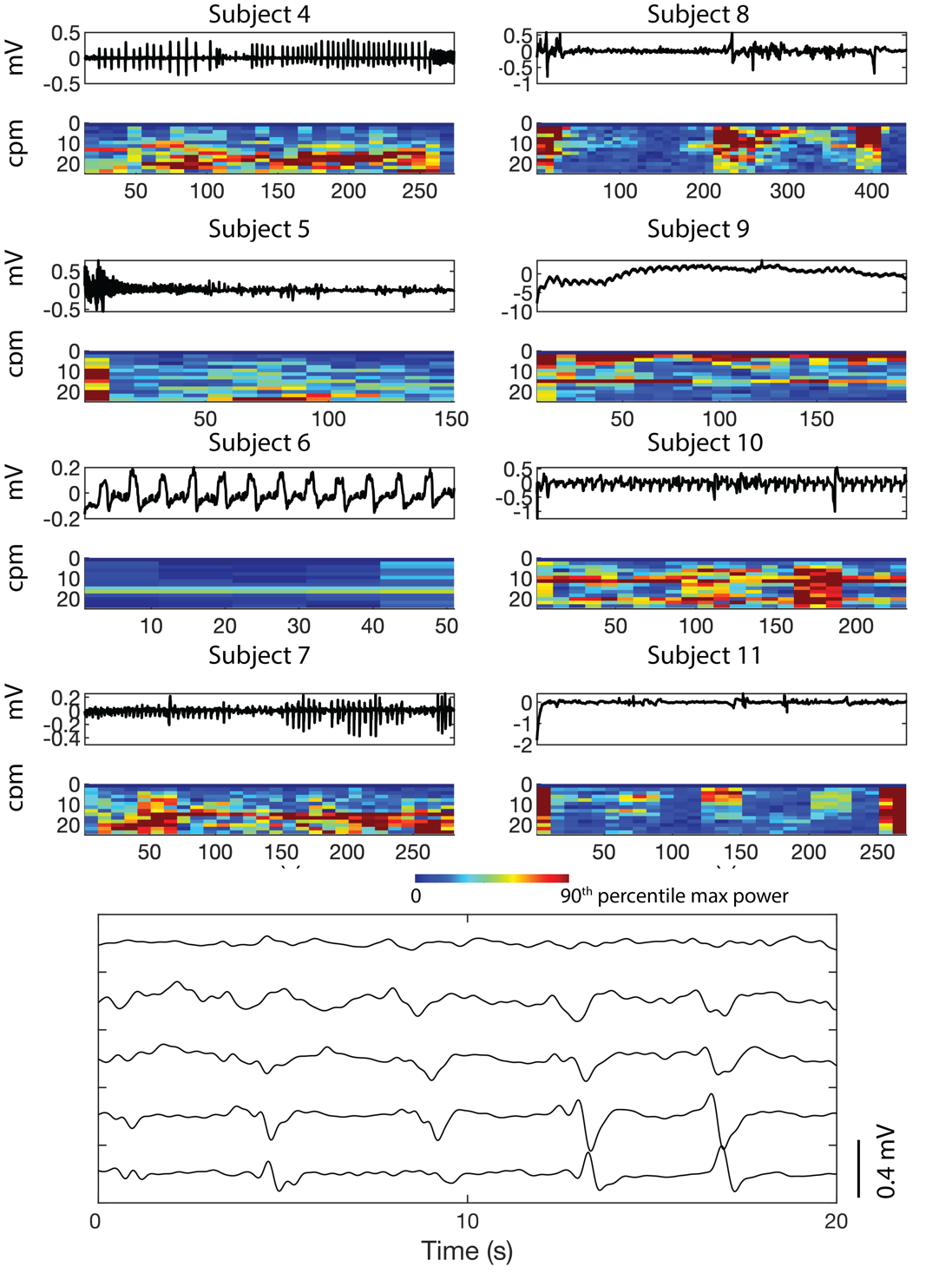


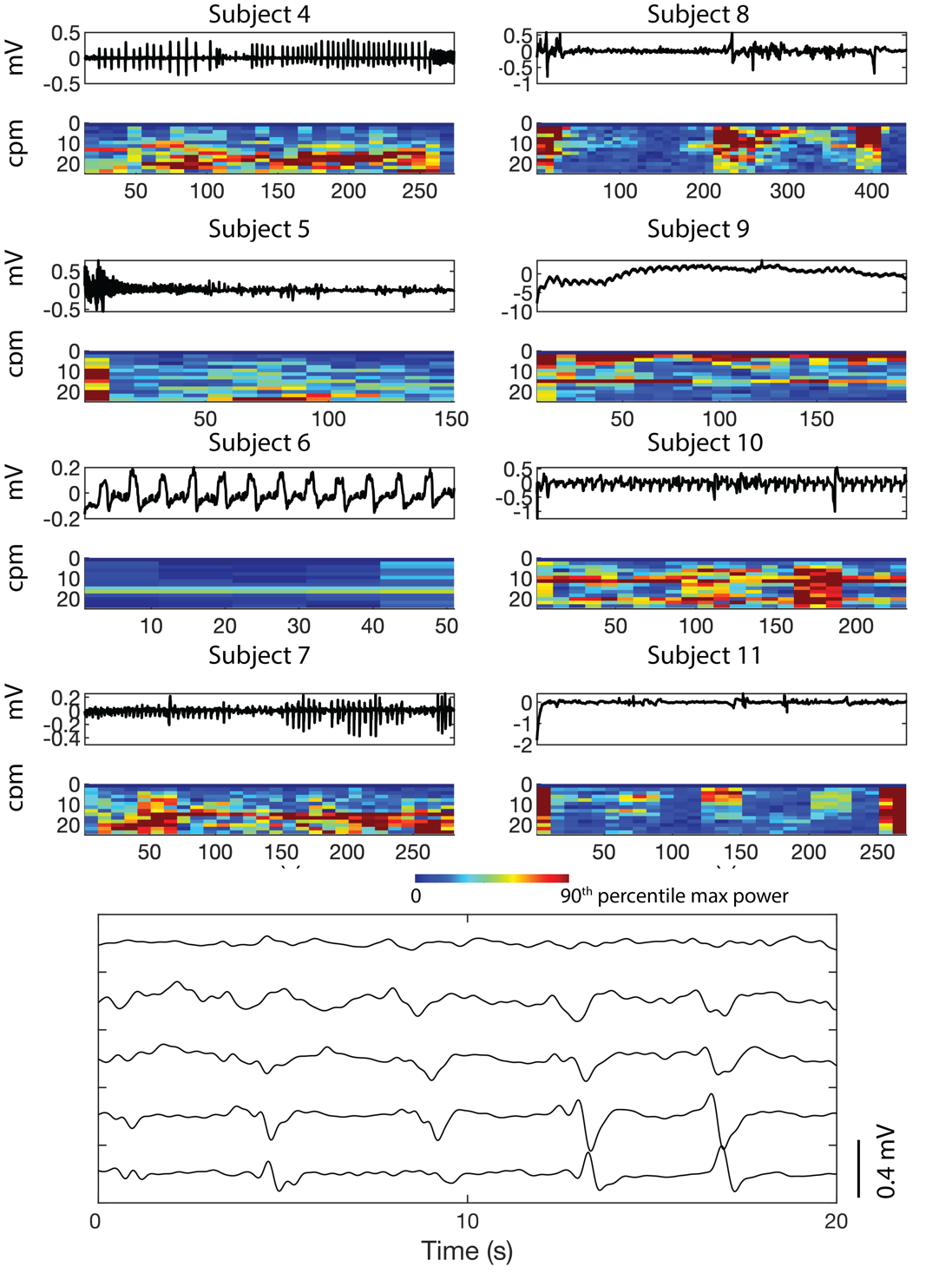


**Additional file 1: Figure S1:** Patient 7 with ~15 cpm, irregular depolarisation activity with apparent propagation and prolonged recovery phase after each initial depolarisation. This was seen in 5-channels over a period of 20 s.

**Additional file 1: Appendix S2: Artefacts**

Recordings of other physiological data were obtained at times, including ventilator and cardiac parameters, according to the descriptors of Paskaranandavadivel et al. (31,32). The frequencies of these activities were consistent with the physiological range recorded on the anaesthetic monitor. **Figure S2** shows several examples of these activities.


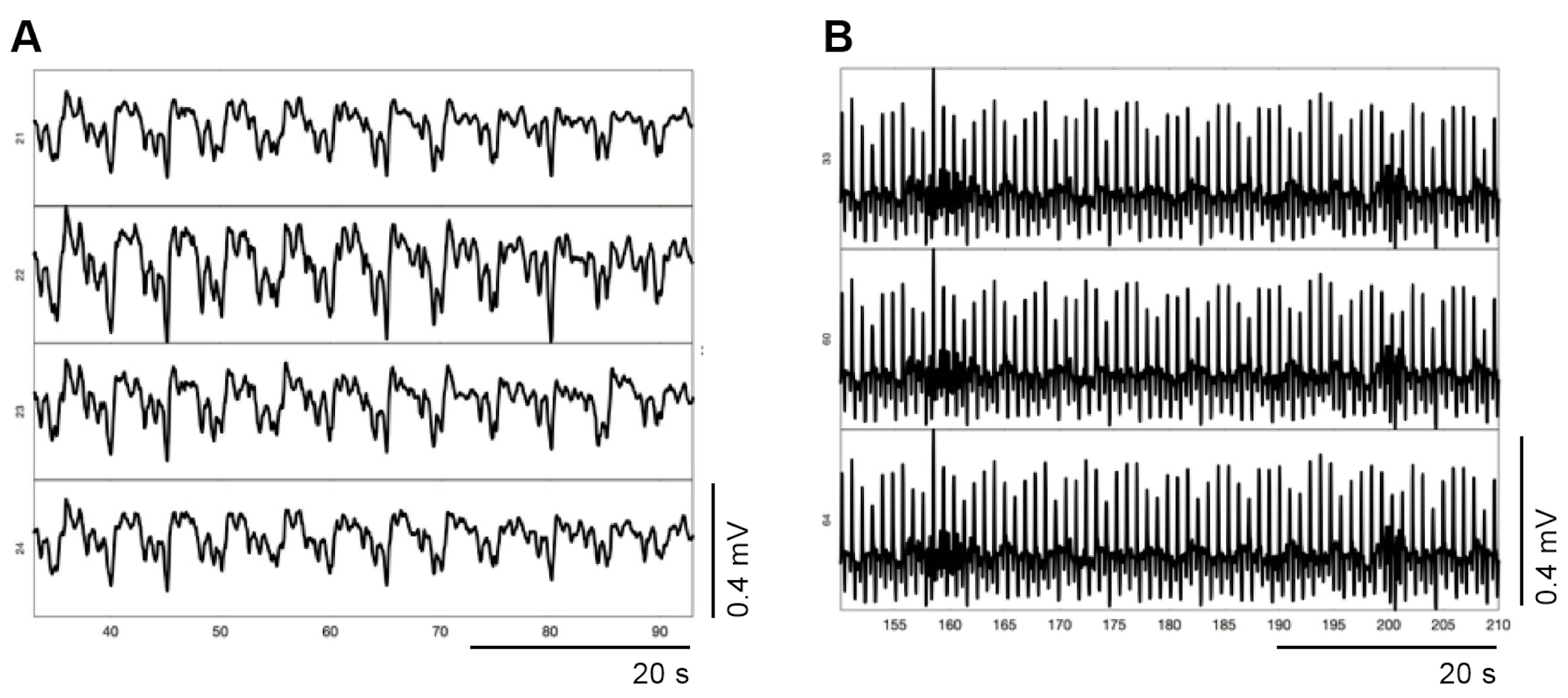


**Additional file 1: Figure S2:** Artefacts obtained on recordings. (A) Ventilator artefacts. (B) Cardiac activity.
